# Accelerated Cardiac Magnetic Resonance Imaging in the Mouse Using an Eight-Channel Array at 9.4 Tesla

**DOI:** 10.1002/mrm.22605

**Published:** 2010-08-25

**Authors:** Jürgen E Schneider, Titus Lanz, Hannah Barnes, Lee-Anne Stork, Steffen Bohl, Craig A Lygate, Roger J Ordidge, Stefan Neubauer

**Affiliations:** 1 British Heart Foundation Experimental MR Unit (BMRU), Department of Cardiovascular Medicine, University of OxfordOxford OX3 7BN, United Kingdom; 2 Rapid Biomedical GmbHRimpar, Germany; 3 Centre for Advanced Biomedical Imaging, University College LondonLondon, United Kingdom

**Keywords:** MRI, phased array, mouse hearts, cardiac function, infarct size, *T*_1_-relaxation times

## Abstract

MRI has become an important tool to noninvasively assess global and regional cardiac function, infarct size, or myocardial blood flow in surgically or genetically modified mouse models of human heart disease. Constraints on scan time due to sensitivity to general anesthesia in hemodynamically compromised mice frequently limit the number of parameters available in one imaging session. Parallel imaging techniques to reduce acquisition times require coil arrays, which are technically challenging to design at ultrahigh magnetic field strengths. This work validates the use of an eight-channel volume phased-array coil for cardiac MRI in mice at 9.4 T. Two- and three-dimensional sequences were combined with parallel imaging techniques and used to quantify global cardiac function, *T*_1_-relaxation times and infarct sizes. Furthermore, the rapid acquisition of functional cine-data allowed for the first time in mice measurement of left-ventricular peak filling and ejection rates under intravenous infusion of dobutamine. The results demonstrate that a threefold accelerated data acquisition is generally feasible without compromising the accuracy of the results. This strategy may eventually pave the way for routine, multiparametric phenotyping of mouse hearts in vivo within one imaging session of tolerable duration. Magn Reson Med, 2010. © 2010 Wiley-Liss, Inc.

Genetically and surgically modified mice are widely used as models for human cardiac disease. MRI has become a routine tool in many research laboratories to noninvasively assess ventricular volumes and mass in order to characterize global cardiac function in these animal models. Analogous to the human heart, MRI can also be used in murine hearts to assess regional and transmural wall motion (1,2), strain (3,4), perfusion (5,6), and infarct size (7,8). Because of the miniature size of murine hearts (∼100 mg) and heart rates of 400–600 beats per minute (bpm) observed even in anesthetized mice, an optimized set up including radio frequency (RF) coils is essential to achieve maximum signal-to-noise ratio (SNR) and to obtain sufficient spatial and temporal resolution. Cardiac MR (CMR) exams in mice are typically performed at ultrahigh magnetic fields ≥ 7 T, using surface- (9–11), volume-type (12–14) RF coils, or a combination of both (6,15). However, dedicated coil arrays consisting of multiple smaller but more sensitive receive coils can provide superior SNR performance compared to volume coils with matching inner diameters. The gain in SNR may be used to improve spatial and/or temporal resolution or to decrease the number of averages required (and therefore scan-time) in an MR scan. Where SNR is sufficient in nonaveraged experiments, accelerated acquisition methods (“parallel imaging”) such as SENSitivity Encoding (SENSE) (16) or GRAPPA (17) may be employed to reduce the scan time further. We have recently shown that it is feasible to complete an exam of global cardiac function in rats at 9.4 T using a four-element array and TGRAPPA (18) within less than 3 min without impaired spatial and temporal resolution (19). Importantly, the accuracy of cardiac functional parameters obtained from these data was maintained using a fourfold accelerated acquisition compared with the unaccelerated case (19). Compared to rat CMR, murine experiments are performed with RF coils of much smaller inner diameter. Smaller geometries represent substantial technical challenges for designing efficient, mutually decoupled coil arrays at ultrahigh magnetic field strengths, and only very few studies have reported on the use of two- to three-element arrays for murine cardiac applications so far (20–22).

We have designed and characterized an eight-channel volume receive array integrated into a linear driven volume transmit birdcage resonator (23). Following the evaluation of the array's SNR performance and its comparison to a quadrature-driven birdcage transmit/receive resonator with identical inner diameter, we hypothesized that the gain in SNR close to the coil array would facilitate accelerated cardiac MRI in mice. The use of (two-element) coil arrays and the feasibility of parallel imaging in cine-mode only has been reported for mouse hearts in vivo so far (22,24), a systematic validation, however, of this technique and combination with other CMR protocols is still lacking. The aim of this study therefore was to investigate the benefits of the eight-channel coil array for murine CMR by combining parallel imaging with global cardiac function imaging (TGRAPPA), with in vivo relaxometry and with contrast-enhanced, high-resolution anatomical imaging in three-dimensional (3D) to measure infarct size. We show that an acceleration factor of 3 is generally achievable without compromising measurement accuracy. Furthermore, the benefit of accelerated imaging in mice is demonstrated using stress cine MRI as it allowed for the first time full heart coverage under intravenous infusion of dobutamine.

## MATERIALS AND METHODS

### RF Coils

For our experiments we used a volumetric coil array consisting of eight elements (id 35 mm, length 32.5 mm) arranged inside a linearly driven volume coil (id 67 mm, length 82 mm/od 115 mm) (see Ref. 23 for details). Comparative experiments were performed using a quadrature driven birdcage coil (id 35 mm, length 35 mm), optimized in geometry and loading for cardiac MR in mice. The maximum possible sample size was 33 mm in both cases.

### MR System

Imaging experiments were carried out on a 9.4 T (400 MHz) MR system (Varian Inc., Palo Alto, USA) comprising a horizontal magnet (bore size 210 mm), a VNMRS Direct Drive™ console and two actively shielded gradient systems (600 mT/m, rise time 180 μsec, od 205 mm, id 120 mm used with the eight-channel array; 1000 mT/m, rise time 130 μsec, od 115 mm, id 60 mm, used for the quadrature-driven birdcage resonator).

### Animal Preparation

C57BL/6 mice were obtained from a commercial breeder (Harlan, UK) at least 1 week prior to the first imaging time point to allow naturalization to new surroundings. The mice were kept under controlled conditions for temperature, humidity and light, with chow and water available ad libitum. Anesthesia was induced in an anesthetic chamber using 4% isoflurane in 100% oxygen. Animals were then positioned prone on dedicated mouse cradles and maintained at 1.5–2% isoflurane at 2 L/min oxygen flow throughout the MRI experiments. Temperature was maintained at ∼37°C using a warm air blanket placed on the front or back of the animal. Cardiac and respiratory signals were continuously monitored using an in-house developed electrocardiogram (ECG) and respiratory gating device (25).

All investigations conformed to Home Office Guidance on the Operation of the Animals (Scientific Procedures) Act, 1986 and to institutional guidelines.

### Global Cardiac Functional Imaging

#### MRI

Five male mice were subjected to cine MRI longitudinally at three sequential time-points (TP) over a period of ∼3 months during which body weight (BW) ranged from 17.1–28.5 g (TP1: 18.3 ± 0.9 g; TP2: 22.8 ± 0.9 g; TP3: 26.9 ± 1.0 g; mean ± SD). These scans were performed to study the effect of increasing coil loading on the coil (array) performance. Each TP consisted of two imaging days, with cardiac functional imaging using the eight-channel array on day 1 and the quadrature birdcage coil on day 2, respectively. After positioning the mice in the magnet with the heart in the centre, and scouting for long- and short-axis orientation of the heart using a cardiac triggered and respiratory gated, segmented gradient-echo sequence, shimming and pulse calibration were performed automatically prior to each experiment. Eight contiguous cine slices (slice thickness slth = 1 mm) were then acquired in short-axis orientation covering the entire heart. The imaging parameters were: field of view (25.6 mm)^2^, matrix size 256 × 256, echo time/pulse repetition time = 1.7/4.6 msec, 15° sinc excitation pulse, number of averages [i.e. number of transients (NT)] = 1 (coil array)/NT = 2 (birdcage coil). The sequence in both cases was ECG-triggered and respiratory gated with steady-state maintenance during respiration (25). The number of frames per cardiac cycle was determined by the heart rate and the number of phase-encoding (PE) steps per respiration cycle—acquired in a segmented fashion (14)—and were adapted to the respective respiratory rate. Additionally, an unaveraged dataset of a midventricular slice was acquired in case of the birdcage coil. For noise-correlation and SNR measurements, identical datasets with the excitation pulse turned off were acquired at the end of the experiment for both birdcage coil and eight-channel array. The total experimental time was approximately ∼40 min for the eight channels and ∼60 min for the birdcage coil, including experimental preparation.

#### Data Analysis

Data reconstruction and analysis were performed off-line using purpose-written *idl*-software (ITT, Boulder, USA) as described previously (19). In brief, in addition to the birdcage-coil and the phased-array (eight coils combined) datasets, twofold and threefold accelerated datasets (*R* = 2, 3) were generated in postprocessing from the phased-array data, followed by TGRAPPA and sum-of-square reconstruction, resulting in four datasets per mouse. All raw data were isotropically zerofilled by a factor of two and filtered [modified third order Butterworth filter (26)] prior to Fourier transformation resulting in an in-plane voxel size of 50 × 50 μm^2^ (experimental resolution: 100 × 100 μm^2^). For each dataset, cardiac structural [i.e., left ventricular mass (LVM); end diastolic volume (EDV); end systolic volume (ESV)] and functional parameters were determined by a single operator (LAS), blinded to animal ID and acquisition/reconstruction scheme, as described before (14), using Amira 4.1 (Visage Imaging GmbH, Berlin, Germany). Intraobserver variability was determined as a measure of reproducibility for volume and TGRAPPA, *R* = 3 datasets by blinded reanalysis. *R* = 4 datasets were generated identically for display purposes only.

SNR measurements were performed for birdcage coil and eight-channel array (for acceleration factors *R* = 1–3) on the fourth frame (corresponding to early systole) of a midventricular short-axis slice using a bootstrap method (27,28) as described in (19). In the unaccelerated, phased-array datasets, SNR was additionally calculated as a function of the number of contributing coils.

### Stress-Cine MRI

Stress-cine MRI was performed in three female mice (28.2 ± 1.9 g) using a threefold accelerated TGRAPPA sequence (*R* = 3) as described above with a matrix size of 128 × 128, applied over two cardiac cycles. Following a baseline scan, dobutamine was infused intravenously via the tail vein at a rate of 27.8 μL/(g·h). Cine MRI commenced ∼7 min after start of infusion, which is the maximal time to achieve steady state in this strain (29). Left-ventricular volumes of all frames were assessed semi-automatically as before. Time–volume curves, normalized to the EDV, of baseline and stress scans were subjected to a Fourier analysis (30,31) using four harmonics to obtain maximum rates of volume change as a measure of contraction and relaxation i.e., (d*V*/d*t*)_min/max_·EDV^−1^.

### *T*_1_ Mapping

*T*_1_ mapping was performed in three female C57Bl/6 mice (33 ± 5 g) as described previously (8,14). In brief, a cardiac gated, segmented SNAPSHOT–FLASH inversion recovery sequence (matrix size = 128 × 128 mm^2^, field of view = 30 × 30 mm^2^, slth = 1 mm, eight PE-steps per segment, echo time/pulse repetition time = 1.6/3.3 msec, pulse repetition time (seg) = 120–150 msec—depending on cardiac cycle length), acceleration factors *R* = 1, 2, 3, and 4 was applied in short-axis orientation. Twenty to thirty images on the inversion curve were used to map *T*_1_. Undersampled datasets were subjected to GRAPPA reconstruction using 24 autocalibration (ACS) lines acquired without inversion pulse. The inversion times, which depended on the heart rate, were logged during the experiment and the mean inversion time for each image on the inversion curve was calculated.

### Infarct Size Measurement

A cardiac triggered and respiratory gated 3D-gradient-echo sequence with *T*_1_-weighting and steady-state maintenance was applied in three male mice (29.3 ± 2.1 g) 1 day post myocardial infarction as reported previously (8) [sequence parameters: matrix size = 256 × 192 × 32, field of view = 30 × 30 × 16 mm^3^, slab thickness 12 mm, PE/seg = 8, echo time/pulse repetition time = 2.0/3.9 ms, pulse repetition time (seg) ∼700 msec, NT = 1, flip-angle = 15°, inversion time individually adjusted in multiples of cardiac cycle lengths (400–420 ms)]. After an intraperitoneal bolus injection of 0.5 μmol/g Omniscan® (Gadodiamide, GE Healthcare/Amersham—total injection volume: 100 μL), the mice were placed in the scanner. Following scouting, shimming and pulse calibration, an ACS dataset (matrix size = 256 × 32 × 16, same imaging parameters otherwise) was acquired. High-resolution infarct imaging started 18 ± 9 min post injection with undersampling factors *R* = 1–4 (applied in random order) in the first phase-encoding direction. The undersampled data were subjected to GRAPPA reconstruction (32). Infarct sizes were measured by a single operator in all datasets (SB—blinded to animal ID and acquisition/reconstruction scheme).

### Statistical Analysis

Agreement in LV mass, EDV, ESV, and EF between the birdcage coil and accelerated and unaccelerated eight-channel array datasets was statistically assessed using ANOVA for repeated measures in IGOR Pro (WaveMetrics Inc., Portland, USA). Agreement in *T*_1_ values and in infarct sizes between the accelerated and unaccelerated datasets was similarly assessed. A *P* value of 0.05 was considered statistically significant.

## RESULTS

**FIG. 1:**
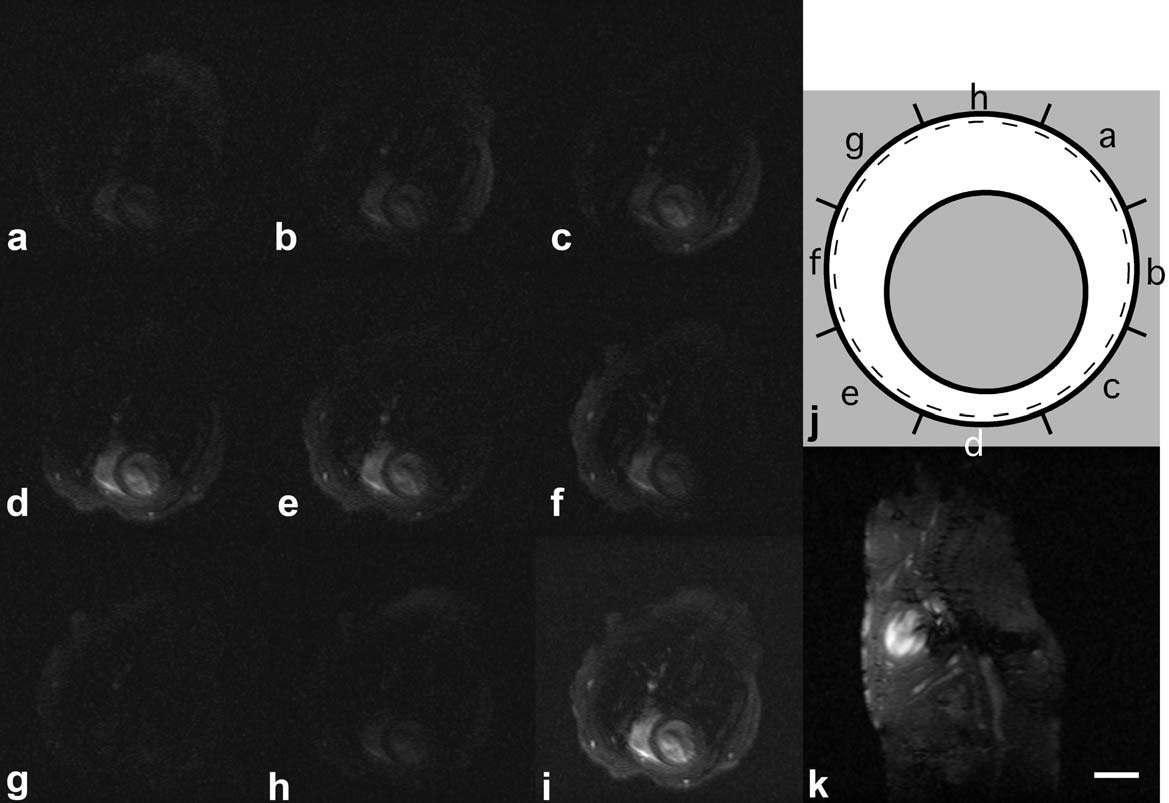
**a–h**: Fully encoded axial *in vivo* gradient echo images of the individual coil elements. The sum-of-squares reconstruction of (**i**) axial and (**k**) sagittal coil images illustrate excellent sensitivity to cover the entire heart in each relevant direction for off-centered positioned mouse. The schematic in panel j depicts the location of the individual coil elements (shown in Fig. 1a–h) relative to the mouse (indicated by the grey circle); the dashed black line represents the inner tube of the probe head, and the solid black circle illustrates the coil-array. Scale bar: 5 mm.

Figure 1a–h shows axial, end-diastolic cardiac triggered and respiratory gated gradient echo images of a mouse heart in vivo, obtained from the individual coil elements of the array (one average). The sum-of-squares reconstructions for the axial and the sagittal view are depicted in panels 1i and 1k, illustrating excellent sensitivity in covering the entire heart in each direction. The scale bar corresponds to 5 mm. The anterior coil elements (Fig. 1b–f) contribute most to the signal in the heart region due to the off-center position of the mouse heart as indicated schematically in panel 1j.

**FIG. 2:**
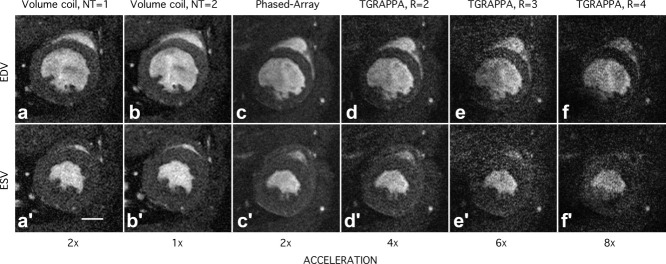
Midventricular end diastolic (top row) and end systolic frames (bottom row) out of cine trains of 27 images in short-axis orientation. The data were acquired with a quadrature birdcage coil—(**a, a**′) one or (**b, b**′) two averages, and (**c, c**′) with the array—sum-of-square reconstruction, one average. From the dataset acquired for Fig. 2c, c′, accelerated datasets with (**d, d**′) *R* = 2, (**e, e**′) *R* = 3 and (**f, f**′) *R* = 4 were generated, followed by a TGRAPPA reconstruction. The pixel size: 50 × 50 μm^2^ in-plane; slice thickness: 1 mm. Scale bar: 2 mm.

Cine imaging was performed longitudinally in order to study the effect of increasing coil loading on the coil (array) performance. Only small changes in body weight between day 1 and day 2 were noted for all imaging time points (ΔBW_TP1_ = −0.5 ± 0.1 g; ΔBW_TP2_ = 0.1 ± 0.3 g; ΔBW_TP3_ = 0.6 ± 0.3 g—day 1 − day 2; mean ± SD), which were significant for TP1 and TP3 (*P* < 0.01 each). Hence, the loading was equivalent for both coils for each time point. Midventricular end-diastolic (top row) and end-systolic frames (bottom row) in short-axis orientation are shown in Fig. 2 for TP3. The data were acquired with the quadrature birdcage coil—one (Fig. 2a, a′) or two (Fig. 2b, b′) averages and with the coil array—sum-of-square reconstruction, one average (Fig. 2c, c′). From the dataset acquired for Fig. 2c, c′, undersampled datasets with (2d, d′) *R* = 2, (2e, e′) *R* = 3 and (Fig. 2f, f′) *R* = 4 were generated, followed by a TGRAPPA reconstruction. The examples shown have been cropped for display purposes, and reflect image quality routinely obtained at all three time points. The two-fold accelerated data (Fig. 2d, d′) show an approximately equivalent image quality compared with the volume coil data, acquired without averaging (Fig. 2a, a′). Epicardial and endocardial border can still be identified in the threefold undersampled images (Fig. 2e, e′).

**FIG. 3:**
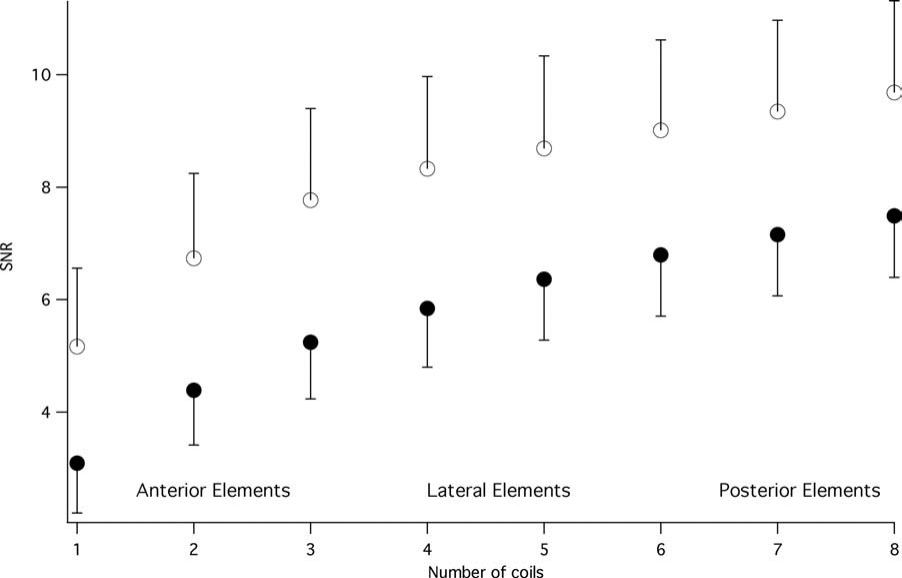
Plot of the mean SNR in anterior (○) and posterior (•) ROI of LV myocardium as a function of number of coils considered for image reconstruction. Coils one to three are the anterior located elements, and, therefore contribute most to the image intensity (mean ± SD, *n* = 5).

To determine the relative contribution of each coil element to improving SNR, the mean SNR as measured in the anterior and the posterior wall is plotted as a function of the number of contributing coil elements (nonaccelerated, sum-of-square reconstruction) in Fig. 3 for TP3. Although the main contribution arose from the three anterior coil elements for both compartments, all eight coil elements increase the SNR. Specifically, an SNR increase (calculated as (SNR_(*n*+1)_ – SNR_*n*_)/SNR_*n*_ × 100%; *n* is the number of contributing coils) of 30% and 15% was observed for the region of interest (ROI) in the anterior LV wall when combing the signal of two or three anterior located coil elements (for the ROI in the posterior wall, the corresponding increase was 42% and 20%, respectively). The two lateral elements increase the SNR in the posterior wall by 11% (four contributing coils in total) and by 9% (five coils in total), whereas the SNR in the anterior wall is only improved by 7% and 4%. The posterior coil elements add another ∼5% to the posterior ROI and ∼4% to the anterior ROI. Similar findings were obtained for the other two time points (data not shown).

**FIG. 4:**
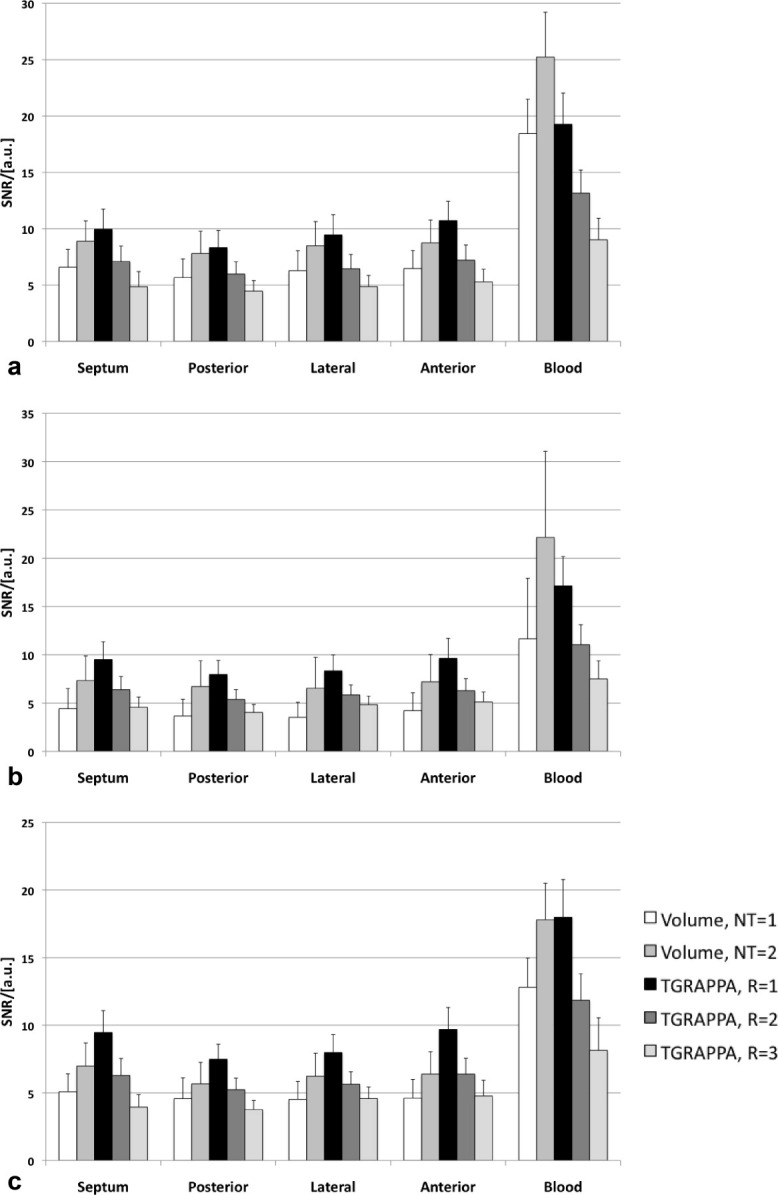
SNR quantification for volume and phased array (accelerated and unaccelerated) in five ROIs of the left ventricle, obtained from a midventricular, cine-frame in early systole. The different panels reflect different mouse body weights (and therefore loading conditions). **a**: BW = 18.3 ± 0.9 g; (**b**) BW = 22.8 ± 0.9 g; (**c**) BW = 26.9 ± 1.0 g (*n* = 5, mean ± SD).

The mean SNR based on location of compartment, coil/acquisition scheme was compared longitudinally under a range of loading conditions. Fig. 4a–c shows bar plots of the mean SNR obtained in the five compartments for each acquisition/reconstruction scheme at each time point. While the quadrature birdcage provided a reasonably homogeneous SNR over the myocardium of the left ventricle, in all data acquired with the coil array, the SNR in the anterior wall was highest and was lowest in the posterior wall as expected. Importantly, the array provides substantially improved SNR performance compared with the quadrature birdcage in all compartments, except for LV blood. The increase in bodyweight between TP1 and TP3 lead to a greater reduction in SNR for the birdcage coil (one average; −26 ± 5%) compared with the array (−10 ± 4%, i.e., 2.6-fold difference between the quadrature birdcage and the array). The ratio of SNR in lateral wall and septal wall combined for quadrature birdcage coil (two averages) to array (one average) was 0.90 ± 0.26 at TP1, 0.78 ± 0.36 at TP2, and 0.76 ± 0.25 at TP3, respectively. The mean *g*-factor, calculated for the GRAPPA reconstruction according to (33), in the cardiac region, averaged over all mice and time points was for *R* = 2 – ḡ = 1.11 ± 0.15 and for *R* = 3 – ḡ = 1.50 ± 0.36, respectively. The average noise correlation between coils ranged between 10% (TP1) and 12% (TP3) (overall mean—11 ± 8%; min—1%; max—29%).

**FIG. 5:**
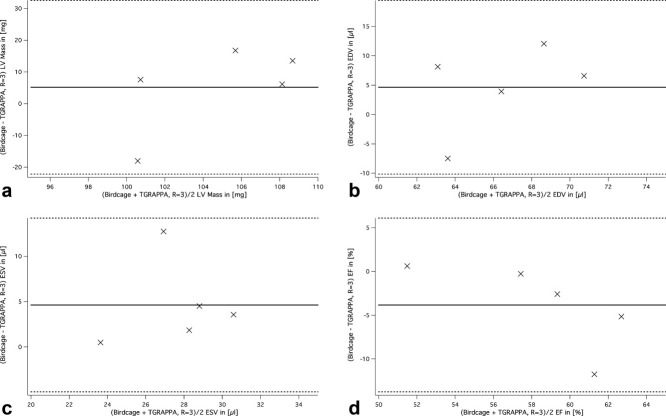
Bland-Altman plots for (**a**) LV mass, (**b**) EDV, (**c**) ESV, and (**d**) EF, obtained from volume coil (2 averages) and *R* = 3, TGRAPPA datasets. The central line on each graph represents the mean of differences between the datasets, while the two flanking solid lines represent ±2 SD.

Cardiac functional parameters and LV mass measurements obtained by a single blinded observer for TP3 are summarized in Table 1. There was no statistically significant difference between measurements for any of the functional parameters. Quantitative analysis was highly reproducible with low variability (overall intraobserver variability 6 ± 6% for both, birdcage coil and TGRAPPA, *R* = 3 datasets; intervariability assessed in three randomly selected datasets: 4.5 ± 3.2 %). Bland-Altman plots for LV mass, EDV, ESV and EF obtained from the birdcage coil compared with threefold accelerated data are shown in Fig. 5a–d. While all data points were contained within the ±2 SD range, there was a positive bias for LV mass (5.2 mg), EDV (4.7 μL) and for ESV (4.6 μL) indicating that volumes measured from the birdcage coil data were larger than those obtained from the TGRAPPA, *R* = 3 datasets. There was a small negative bias for EF (−3.8%).

**Table 1:** Left Ventricular Mass and Functional Parameters for TP 3 (Mean ± SD)

	Birdcage coil	Phased array	TGRAPPA, *R* = 2	TGRAPPA, *R* = 3
*n*	5	5		
Body weight (g)	27.2 ± 0.9	26.6 ± 1.0		
HR (bpm)	445 ± 27	458 ± 40		
LV mass (g)	107.4 ± 9.8	100.5 ± 6.0	100.9 ± 5.6	102.2 ± 5.4
EDV (μL)	68.8 ± 6.0	66.7 ± 3.9	66.4 ± 5.5	64.2 ± 3.5
ESV (μL)	30.0 ± 3.7	27.2 ± 2.4	28.5 ± 1.9	25.3 ± 3.3
SV (μL)	38.9 ± 3.5	39.6 ± 4.5	37.8 ± 5.1	38.8 ± 5.4
EF (%)	57 ± 3	59 ± 4	57 ± 4	60 ± 6
CO (mL/min)	17.3 ± 2.3	18.0 ± 1.8	17.3 ± 2.2	17.8 ± 3.0

**FIG. 6:**
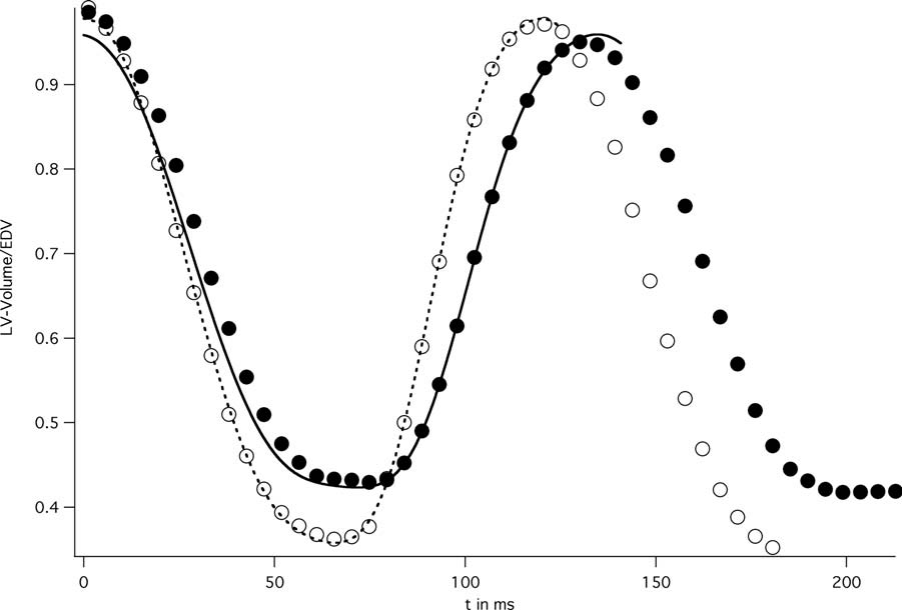
Representative time volume curves for the entire left ventricle, normalized to the end diastolic volume. The volumes were obtained from threefold undersampled TGRAPPA cine data acquired over two cardiac cycles under baseline conditions (“•” symbols) and under intravenous dobutamine infusion (“○” symbols). Both curves were fitted (lines) to obtain left-ventricular peak-filling and ejection rates.

In a test application for accelerated cine imaging, time–volume curves were obtained from the whole ventricle during the experiment using TGRAPPA, *R* = 3 under baseline conditions and under intravenous dobutamine infusion (Fig. 6). The increase in heart rate (corresponding to a reduction of the RR-interval length) and the change in contractility [i.e., (d*V*/d*t*)_min/max_·EDV^−1^] are evident. More specifically, an increase in heart rate of 11 ± 2% was observed under dobutamine infusion. The maximum rate of volume change (d*V*/d*t*)_max_·EDV^−1^ as a measure of relaxation increased from 20.3 × 10^3^ ± 5.3 × 10^3^ s^−1^ to 22.8 × 10^3^ ± 4.1 × 10^3^ s^−1^, whereas the minimum rate of volume change (d*V*/d*t*)_min_·EDV^−1^ as a measure of contraction decreased from −(14.5 ± 1.7) × 10^3^ s^−1^ to −(17.4 ± 1.7) × 10^3^ s^−1^ (*n* = 3, mean ± SD, *P* = 0.2 and 0.06, respectively). Interestingly, determining the contractility parameters in the same way from a single midventricular slice only showed no difference for (d*V*/d*t*)_max_·EDV^−1^, while (d*V*/d*t*)_min_·EDV^−1^ was significantly different to the values determined for the whole heart (*P* < 0.05).

**FIG. 7:**
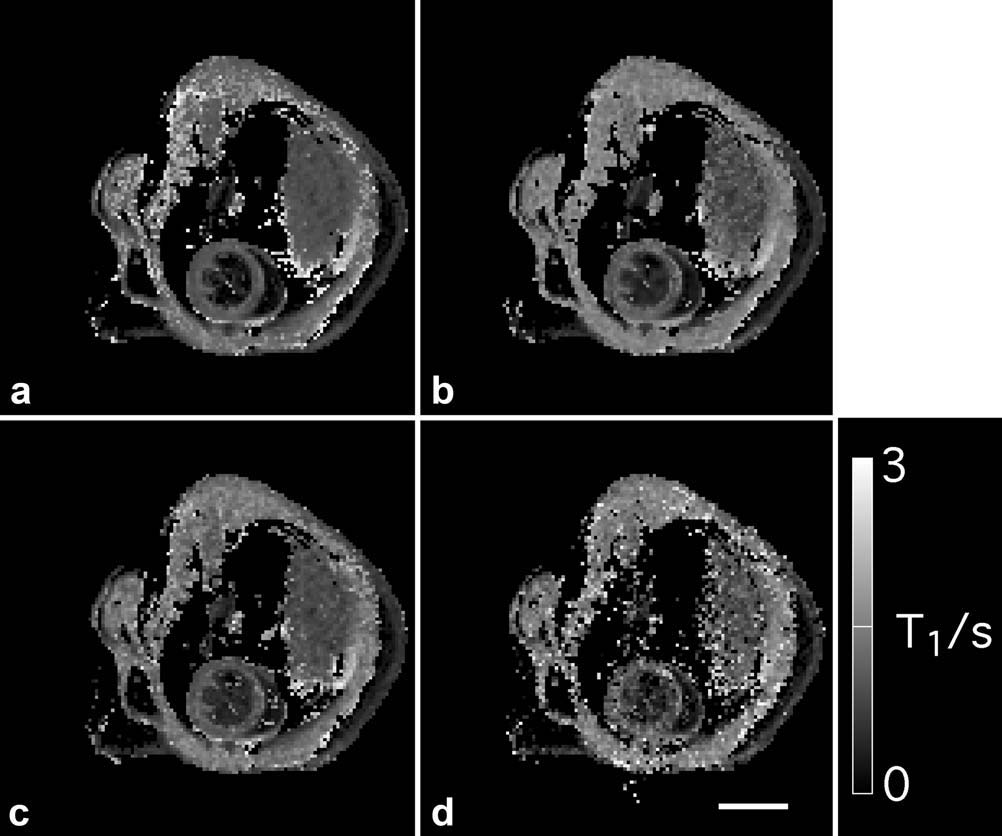
*T*_1_-parameter maps in a midventricular short-axis slice for acceleration factors (**a**) *R* = 1, (**b**) *R* = 2, (**c**) *R* = 3, and (**d**) *R* = 4, respectively. The maps were masked in the range 0–3 sec to remove outliers from the fit. Twenty-four autocalibration lines were used to reconstruct the missing information from the undersampled datasets. *T*_1_-values for various tissue types are listed in Table 2. Scale bar: 5 mm.

To determine whether *T*_1_-mapping and *T*_1_-weighted imaging could also be accelerated, parallel imaging was combined with the respective method in two dimension (2D) or 3D. Representative *T*_1_-parameter maps for acceleration factors *R* = 1–4 are shown in Fig. 7a–d. The *T*_1_ maps were masked in the range 0–3 sec to remove outliers from the fitting procedure. No significant difference in the parameter maps can be seen up to *R* = 3, while the increased noise levels and *g*-factors in the fourfold undersampled inversion recovery data impact on the fit accuracy (Fig. 7d). Quantitative results of the accelerated and non-accelerated *T*_1_-relaxation time measurements obtained from ROIs placed in liver, skeletal muscle of the chest wall, and left ventricular myocardium (at least 100 pixels per ROI) are listed in Table 2. The ANOVA test revealed no statistically significant difference between the different *T*_1_ values for each tissue type.

**Table 2:** *T*_1_ Relaxation Time Measurements (Mean ± SD)

*T*_1_ (s)	*R* = 1	*R* = 2	*R* = 3	*R* = 4
LV Myocardium	1.00 ± 0.07	1.03 ± 0.09	0.99 ± 0.11	1.05 ± 0.19
Skeletal Muscle	1.34 ± 0.10	1.41 ± 0.09	1.38 ± 0.12	1.48 ± 0.18
Liver	1.03 ± 0.06	1.02 ± 0.09	0.98 ± 0.09	1.06 ± 0.16

**FIG. 8:**
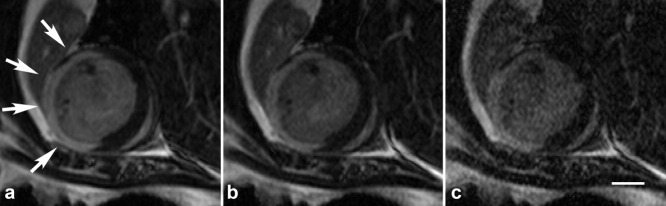
Contrast-enhanced midventricular short-axis slice out of 3D stack for acceleration factors (a) *R* = 1, (b) *R* = 2, and (c) *R* = 3, respectively. The arrows in Fig. 8a indicate the infarcted area. Scale bar: 2 mm.

Midventricular sections out of a 3D slab following intraperitoneal injection of Gd are shown in Fig. 8 for acceleration factors *R* = 1–3. The required scan times (also depending on the ratio heart to respiratory rate) were: *R* = 1: 1189 ± 85 sec; *R* = 2: 626 ± 44 sec; *R* = 3: 401 ± 47 sec; and *R* = 4: 317 ± 40 sec, respectively. Table 3 summarizes the left ventricular volumes, infarct volumes and infarct sizes for the individual mice obtained by blinded analysis. It can be seen that the infarct volumes and, therefore, the infarct sizes decreased significantly with increasing acceleration factors (*P* < 0.01 and *P* = 0.027, respectively), while the left ventricular volumes remained constant (*P* = 0.73).

**Table 3:** Volume and Infarct Size Assessment

LV volumes in μL				
Infarct volumes in μL				
Infarct sizes (IS)	*R* = 1	*R* = 2	*R* = 3	*R* = 4
Mouse 1	79.8	81.0	84.9	86.3
	38.9	32.4	31.8	30.8
	48.8%	40.0%	37.5%	35.7%
Mouse 2	75.6	76.7	77.5	75.7
	40.0	40.5	34.6	30.6
	52.9%	52.7%	44.6%	40.5%
Mouse 3	78.1	75.4	77.6	67.0
	35.3	32.8	31.1	28.1
	45.2%	43.5%	40.1%	42.0%
Mean IS ± SD	49.0 ± 3.8%	45.4 ± 6.6%	40.8 ± 3.6%	39.4 ± 3.3%

## DISCUSSION

The aim of this article was to establish and validate a phased-array setup for cardiac MRI in mice at ultrahigh magnetic fields (i.e., 9.4 T). We used an eight-channel array designed to fit mice with a body weight range of approximately 20–35 g, and compared the phased array to a quadrature driven birdcage coil with a matching inner diameter that has been optimized and is routinely used in various laboratories for cardiac mouse MR. While the coil setup has been characterized in detail previously (23), the applicability for CMR needed to be determined. As is typical for the concept of a transmit body coil combined with a receive array, the transmit efficiency of the volume transmit coil is low compared with a quadrature Tx/Rx birdcage, due to the very low filling factor of the Tx resonator and the missing quadrature polarization. The inversion recovery experiments required a six times longer inversion pulse at the same power level than for the quadrature birdcage in order to completely invert the spins. Furthermore, the longitudinal homogeneity of the volume transmit coil is affected by the presence of the receive array, which shields the sample against the Tx amplitude of (excitation) radiofrequency field. This causes some amplitude of (excitation) radiofrequency field inhomogeneity in long-axis of the coil, and can impact on the automated RF pulse calibration, which was performed over an axial, 10-mm-thick slice containing the entire heart at the beginning of each experiment. The reduced SNR in the blood-pool of the LV for the array compared to the birdcage coil is due to a lower flip angle and therefore reduced inflow effect. The flip angle miscalibration will also inevitably affect the quantitative SNR measurements (Fig. 4), which were estimated to be in the order of 10% lower for LV myocardium and ∼50–60% lower for LV blood. Therefore, while the main conclusion of our validations remains unaffected, the comparison between array and birdcage may be even more favorable toward for the eight-channel array than observed in our studies.

In agreement with our previous work (23), we observed in our longitudinal cine study 2.6-fold larger SNR-reduction for the birdcage coil than for the array with an increase in mouse body weight of ∼10 g (Fig. 4a,c). Furthermore, the coupling between the individual coil elements as assessed quantitatively by the noise correlation matrix was only minimally affected by body weight. On the other hand, the array is substantially more sensitive in close vicinity of the coil elements. To utilize this effect, we placed the mouse slightly rotated in a dedicated animal cradle consisting only of a foil at the body region off-centered in the magnet. The SNR in the heart region is therefore dominated by the three anterior coil elements. Nevertheless, all eight coils are contributing constructively to the SNR in the heart and benefitted particularly the posterior wall of the left ventricle (Fig. 3).

The most important advantage of the array over the birdcage coil is the parallel imaging capability, which provides a significant reduction of scan time. Three different techniques were investigated in this context, i.e., cardiac functional imaging, *T*_1_-mapping and contrast-enhanced infarct MRI.

Similar to our previous study in rats (19), we used TGRAPPA (18) to investigate how accelerated cardiac MR impacted on the accuracy of cardiac functional and structural parameters. All datasets were therefore analysed blinded by a single operator as this technique is well established in our laboratory and our analysts are interchangeable. The use of a matching (birdcage) coil for comparison required experiments to be conducted on two (successive) days. To minimize any effect of the experimental procedure in general, the scans with the eight-channel array were always conducted on day one due to the shorter protocol. LV mass, EDV, and ESV were obtained for all acquisition/reconstruction schemes with high accuracy (Fig. 5 and Table 1). The small bias in ventricular volumes and mass, which is still within the physiological variability, is likely caused by the signal averaging of the volume coil data. Validation experiments on a phantom with known volume showed a difference of <2% between both coils, which is within the accuracy of image segmentation. Furthermore, it cannot be excluded that (minor) differences in gradient scaling and/or physiological setup contributed to the found bias. Only differences within the reproducibility of the segmentation were found when comparing phased-array data without (*R* = 1) and with threefold acceleration (data not shown). There was no difference in parameter variability between high- and low-SNR datasets, importantly despite the decrease in SNR with increasing acceleration factors. In particular, the overall intraobserver variability and interobserver variability for both volume and array coil data were in the same range as previously published values (12,14). In agreement with our previous work, this demonstrates that even relatively low-SNR data are sufficient to accurately determine cardiac functional parameters in mice, and that sacrifice of SNR for parallel imaging is an acceptable trade-off to reduce the acquisition time (19).

One application facilitated by the combination of parallel imaging with cine MRI is pharmacological stress testing, e.g., by dobutamine infusion, and the assessment of LV volumes and maximum rates of volume change using whole ventricular coverage. LV volumes of the entire ventricle following intraperitoneal bolus injection of dobutamine have been quantified before (34), contractile parameters, however, are only typically obtained from a single midventricular slice in short-axis orientation (34,35). As with contrast-enhanced applications (see for example, Ref. 8), intravenous administration has the advantage over intraperitoneal injections of achieving a rapid steady-state response, which is independent of absorption rates. This is particularly the case for dobutamine, which is very rapidly metabolized. In the mouse, the duration of intravenous infusions is limited by small administrative volumes. More specifically, given the small blood volume of a mouse of ∼2 mL, the maximum intravenous administrable volume is 200 μL, before causing adverse physiological effects. This is reached after approximately 15 min in a 28 g mouse and an infusion rate of ∼28 μL/(g h). Allowing for a stabilization period of 5 min, this leaves an imaging window of only about 10 min, which is halved further by imaging over two cardiac cycles. This could only be achieved by reducing the imaging matrix size and acquiring unaveraged, threefold undersampled datasets, which took about 2.5 min for eight short-axis slices. Our pilot study also demonstrated a difference in maximum rate of contraction (i.e., (d*V*/d*t*)_min_·EDV^−1^) between single slice and whole LV volume analysis. While the physiological relevance of this finding is questionable in this particular example (we used normal mice), it may well play a role in surgically or genetically modified hearts, where the exact location and orientation of the midventricular slice may impact on the calculated minimum and maximum rates of volume change. Whole heart coverage eliminates this problem.

*T*_1_ has been quantified in mouse hearts before (5,6,8,14), and typically requires long repetition times to allow the spins to fully relax. We demonstrated that a threefold speedup still provides high-quality *T*_1_ parameter maps (Fig. 7c) and reproducible *T*_1_ values (Table 2), reducing the scan time from ∼2.5 min (*R* = 1) down to ∼50 sec (*R* = 3) for a single slice. Only three heartbeats (<500 msec) were required to acquire the ACS data, required for the GRAPPA reconstruction. Mapping the myocardial blood flow (i.e. perfusion) by means of arterial spin-labeling (ASL) techniques requires two *T*_1_-parameter maps, acquired with slice-selective and with global inversion pulse, respectively. Hence, parallel imaging will reduce the scan time requirements for ASL-based perfusion imaging in mouse hearts below 5 to 10 min, depending on the method used (5,8). Importantly, the ACS lines need to be acquired only once and can be used to reconstruct both parameter maps.

Contrast enhanced infarct size assessment in combination with parallel imaging was performed to investigate the benefits for 3D applications. The short-axis orientation of the imaging slab is dominated by the axial component. Therefore, the datasets were only undersampled in the first phase-encoding direction, as the coil elements are located radially. We found a statistically significant trend toward smaller infarct sizes with increasing *R*. However, a decrease in SNR cannot be responsible for this result alone as the LV volumes were still accurately obtained for *R* = 3 (Table 3). Hence, it cannot be excluded that this variation is also caused by the contrast agent kinetics, despite the acquisition of the datasets with different acceleration factors in random order. This hypothesis is also supported by the fact that the reduced mean infarct size for *R* = 2 compared with *R* = 1 is mainly driven by mouse 1 (Table 3), and that a comparable image quality was obtained for both datasets (as demonstrated for a midventricular slice in Fig. 8a,b). A thorough investigation of this phenomenon would require substantially more mice and a different study design, which is beyond the scope of this study. Importantly, the scan-time requirements for the acquisition of the ACS data can in our case be reduced to four heartbeats (∼500 msec) by turning off the *T*_1_ module, and by fully utilizing the reconstruction proposed by Blaimer et al (32), which eliminates the need for applying phase encoding in the third dimension.

In conclusion, we have validated the use of an eight-channel volume phased-array coil for cardiac MRI in mice at 9.4 T. We have combined 2D and 3D sequences to quantify global cardiac function, *T*_1_ relaxation times and infarct sizes, with parallel imaging techniques. A threefold acceleration was generally feasible without impairment of accuracy of the various parameters. These achievements allowed us to measure for the first time left-ventricular peak filling and ejection rates under intravenous infusion of dobutamine. Our method offers substantial reductions in scan time, without compromising the accuracy of the results. This strategy may pave the way for routine multi-parametric cardiac phenotyping, including stress tests and contrast-enhanced techniques, within a single imaging session of tolerable duration.
